# The Potential Application of Commercially Available Active Video Games to Cardiac Rehabilitation: Scoping Review

**DOI:** 10.2196/31974

**Published:** 2022-03-18

**Authors:** Ryuichi Sawa, Masakazu Saitoh, Tomoyuki Morisawa, Tetsuya Takahashi, Yuh Morimoto, Nobuyuki Kagiyama, Takatoshi Kasai, Birthe Dinesen, Hiroyuki Daida

**Affiliations:** 1 Department of Physical Therapy Faculty of Health Science Juntendo University Tokyo Japan; 2 Department of Digital Health and Telemedicine Research and Development Faculty of Health Science Juntendo University Tokyo Japan; 3 Faculty of Health Science Juntendo University Tokyo Japan; 4 Department of Cardiovascular Biology and Medicine Juntendo University Graduate School of Medicine Tokyo Japan; 5 Laboratory for Welfare Technologies - Telehealth & Telerehabilitation, Sport Sciences - Performance and Technology Department of Health Science and Technology Aalborg University Aalborg Denmark

**Keywords:** active video game, cardiac rehabilitation, physical exercise, rehabilitation, serious games, CVD, AVG, cardiovascular disease, exercise, safety, adherence

## Abstract

**Background:**

Commercially available active video games (AVGs) have recently been used for rehabilitation in some specific patient populations but rarely in those with cardiovascular disease (CVD). Commercially available AVGs are designed to increase motivation for continuous play, which could be applicable to the long-term cardiac rehabilitation process.

**Objective:**

The objective of this scoping review was to assess the effectiveness of AVG-induced physical exercise, safety management, and patient adherence by applying commercially available AVGs to cardiac rehabilitation.

**Methods:**

Four databases (CINAHL, MEDLINE, PubMed, and SPORTDiscus) were searched for all years up to August 12, 2020. Articles were retained if they were written in English, included patients with CVD who were aged 18 years or older, and used AVGs as part of a physical exercise program. The included studies were then evaluated from the viewpoints of effectiveness as physical exercise, safety, and adherence management.

**Results:**

Among 120 nonduplicate articles reviewed, 5 (4.2%) were eligible for inclusion, of which 3 (2.5%) were reported by the same research group. The AVG consoles used were Xbox Kinect and Nintendo Wii, and sports-related programs were adopted for the intervention. No adverse cardiac events occurred in the identified studies, and dropout rates tended to be low.

**Conclusions:**

AVGs appear to be safe and feasible for promoting an active lifestyle in patients with CVD. However, the effectiveness of AVGs alone as a therapeutic exercise to improve physical function may be limited.

## Introduction

Cardiovascular disease (CVD) is the most prevalent noncommunicable disease and the leading cause of death globally [1]. Despite the proven benefits for the improvement in mortality from cardiac rehabilitation and strong guideline recommendations [2-4], a variety of barriers that reduce adherence to cardiac rehabilitation remain, especially after hospital discharge [5-7]. In particular, barriers related to accessibility are expected to become the major barrier with progressive population aging. Symptoms associated with CVD lead to an excessively sedentary lifestyle, and this physical inactivity reinforces the tendency to be trapped in an age-related downward spiral toward frailty and disability [8,9]. Therefore, approaches that interrupt or help prevent falling into this spiral are urgently needed in cardiac rehabilitation.

**Figure 1:**
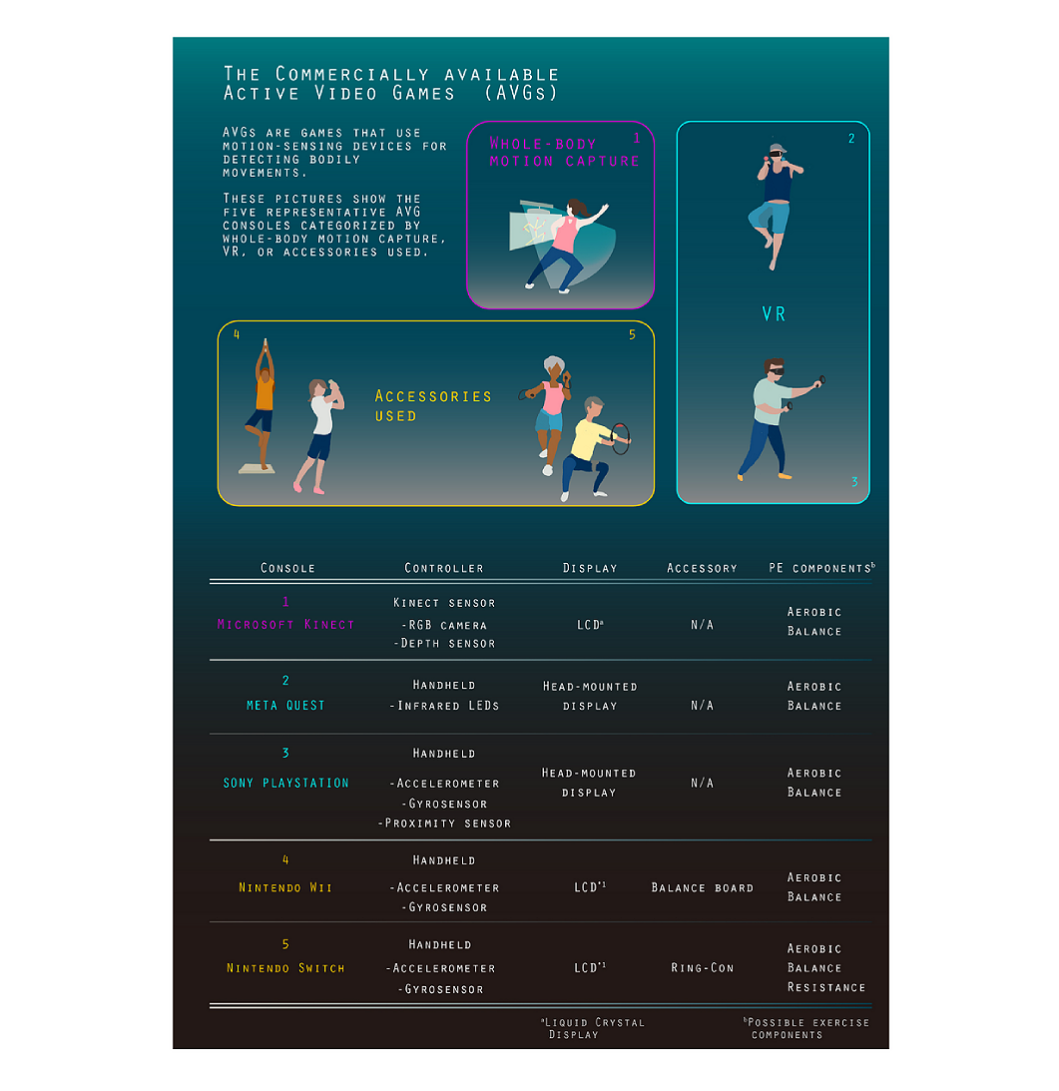
Categories and characteristics of commercially available active video game consoles. LED: light-emitting diode; N/A: not available; RGB: red, green, blue; VR: virtual reality.

Active video games (AVGs), which are games that require players to interact with objects within a virtual environment using some part of their body as the controller [10], have recently emerged because of the evolution of motion-sensing devices that are designed to detect and measure movements. During the past decade, video game companies have launched several commercially available AVG consoles (Figure 1). Owing to their appealing designs, AVGs can increase motivation and long-term engagement with physical exercise in the general population, including older adults [10-13]. A systematic review has revealed the effectiveness of Nintendo Wii in the rehabilitation of adults with stroke [14]. However, only 6 studies were included in the review, suggesting that the evidence is still in the construction stage.

Therapeutic exercise is a core component in cardiac rehabilitation and aims to improve physical function such as exercise capacity and muscle strength through adequate exercise prescriptions [2]. On the other hand, because of the long-term rehabilitation process, practice guidelines consistently recommend comprehensive rehabilitation programs involving multiple components (eg, health education, advice on CVD risk reduction, physical activity, stress management) [3,4,15]. Furthermore, because of the aging population, it has become more important to provide multicomponent exercise programs as well as to increase patient motivation to engage in physical exercise. A previous systematic review reported that no studies integrated commercially available AVGs with multicomponent physical rehabilitation programs for motor function in patients with CVD [16].

Given this background, the objective of this scoping review was to explore the possibility of applying commercially available AVGs to cardiac rehabilitation. With this in mind, we assessed the available evidence in the literature for the physical effects of using AVGs in patients with CVD to examine ways to manage the safety of and adherence to such interventions.

## Methods

### Design

We conducted a scoping review in accordance with the guidelines described by PRISMA-ScR (Preferred Reporting Items for Systematic Reviews and Meta-Analyses Extension for Scoping Reviews) [17].

### Search Strategy

A literature search of the electronic databases CINAHL, MEDLINE, PubMed, and SPORTDiscus was conducted for all years up to August 2020. The literature search was conducted using search terms covering medical and health science terminology (Multimedia Appendix 1).

### Selection Process

Following the removal of duplicates, titles and abstracts were screened by at least 2 independent reviewers (TM, MS, YM, and RS) to exclude irrelevant articles as the first screening step. Articles were included if they focused on the influence of commercially available AVGs on patients with CVD aged 18 years or older. A commercially available AVG was defined as a digital game that video game companies launched into the entertainment market and that have controllers mounting motion-sensing devices for detecting bodily movements as signals. The inclusion criteria were articles that reported original quantitative data including case studies, studies with participants aged 18 years or older, studies that included participants with CVD, and studies that used a commercially available AVG as an intervention tool. Articles were ineligible if they were topic news or an editorial, pictorial, perspective, letter, conference paper, or review. Those that reported qualitative data were also ineligible for this review. The exclusion criteria were studies that used commercially unavailable AVGs (research-based AVGs) and articles published in a language other than English. In the second screening step, the articles included from the first screening step were downloaded and screened again by the same independent reviewers using the same criteria as that used in the first screening. Disagreements arising between the reviewers at any stage of the study selection process were resolved through team discussions or by a third reviewer (TT) to reach consensus on whether the article met the inclusion criteria. Reference lists of relevant articles were also hand-searched to identify additional appropriate articles for inclusion in this review. Review articles were not included in this study, but were checked for potentially relevant references.

### Data Extraction

Using a structured sheet in Excel (Microsoft), data were extracted by 1 reviewer (RS) and subsequently checked for accuracy by the other authors. The extracted data included details about the authors, year of publication, study design, location of the study, aims of the study, sample characteristics, AVG console used in the intervention, AVG programs used in the intervention, details of the AVG intervention (eg, period, frequency, duration, intensity), and the outcome measures related to physical exercise. Physical exercise was defined as bodily movements to enhance or maintain physical activity. Additional data of interest included the study setting, supervision, delivery method for the AVG interventions (AVG alone or AVG with other programs), effectiveness of the AVG in terms of physical exercise, safety management, the number of adverse events, adherence management, the number of patients who dropped out, and reasons for dropout. Safety management was defined as efforts to prevent adverse events during the intervention period. Adherence was defined as performance of the intervention as planned, and efforts to manage patient adherence were extracted as adherence management. These data were grouped and arranged in Excel by the type of setting and the presence of supervision to report the study outcomes.

## Results

**Figure 2:**
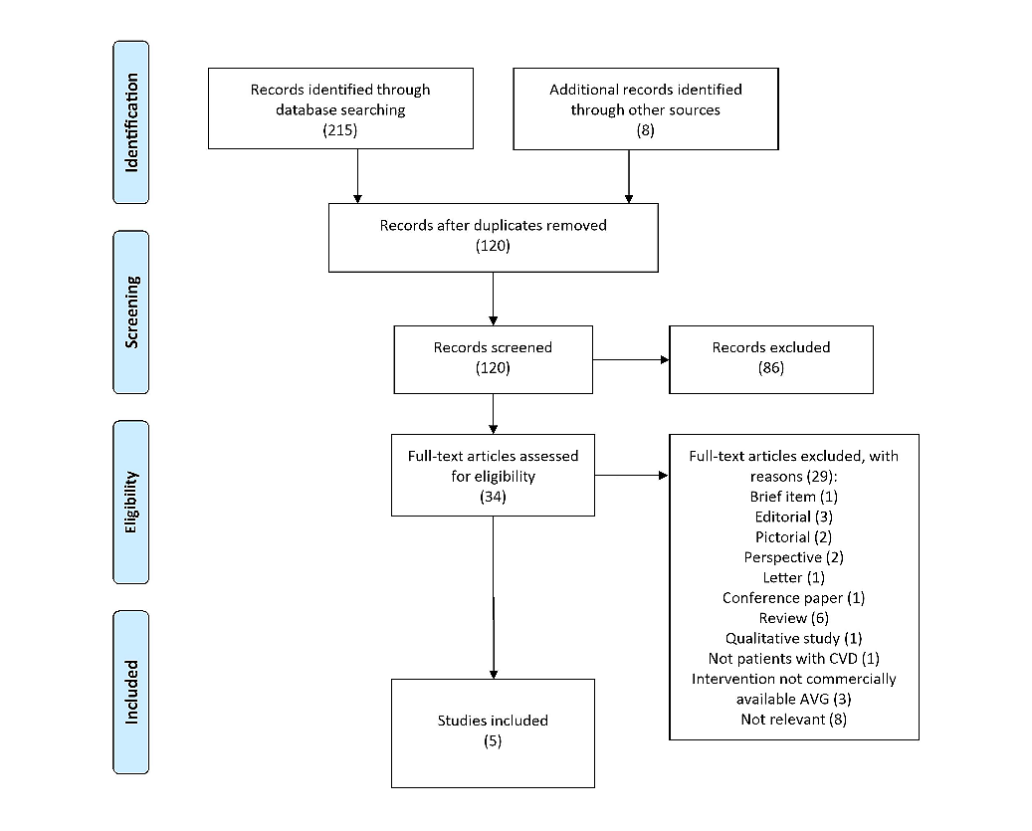
A flow diagram of studies included according to PRISMA-ScR (Preferred Reporting Items for Systematic reviews and Meta-Analyses extension for Scoping Reviews). AVG: active video game; CVD: cardiovascular disease.

A flowchart of the systematic screening process with the number of articles included or excluded at each stage is shown in Figure 2.

From the initial 223 identified articles, 5 (4.2%) were retrieved and screened for eligibility. In terms of study design, 3 of the 5 (60%) articles reported randomized studies. The remaining 2/5 (40%) articles were a case study and a pre-post study. The details of the identified articles are shown in Table 1, and a summary of the intervention delivery modes and effectiveness is shown in Table 2. Table 3 shows the summary of adverse events and adherence across studies. One study investigated the impacts of adding AVG-based physical exercise to cardiac rehabilitation programs on acute hemodynamics in 27 outpatients with CVD or CVD risk [18]. That study was a cluster-randomized crossover trial and used the Xbox Kinect as an alternative tool for warm-up and conditioning sessions in center-based cardiac rehabilitation. Similar physiological acute hemodynamic responses to cardiac rehabilitation were found, with higher magnitudes of heart rate, respiratory rate, and the rate of perceived exertion during and after cardiac rehabilitation with the AVG. Another study conducted in 32 outpatients with CVD in a hospital reported that those who had been allocated into the AVG group showed significant improvements after a 6-week intervention period in physical activity measured as accelerometer arbitrary unit (AAU; median baseline, 255 AAU/min; end of program, 322 AAU/min; *P*=.04), whereas those allocated into the conventional cardiac rehabilitation group showed no change (median baseline, 225 AAU/min; end of program, 247 AAU/min; *P*=.99). Measures of energy expenditure also improved significantly in the AVG group compared with those in the conventional cardiac rehabilitation group [19]. These 2 studies took the exercise intensity of AVGs into consideration based on physiological measures such as heart rate. On the other hand, 3 studies reported by the same research group [20-22] focused on the effects of home-based AVGs in patients with heart failure. The research group has published the results from a case study, a pilot study [22], and an open-label randomized study [20]. A total of 605 patients from multiple countries got the same instructions about the time and frequency for playing AVGs but were given no concrete numerical target regarding exercise intensity. Although the intervention through home-based AVGs was safe and feasible, it was not effective in improving the following outcomes: the 6-minute walk test, unilateral isotonic heel-lift, bilateral isometric shoulder abduction, unilateral isotonic shoulder flexion, exercise motivation, exercise self-efficacy, and physical activity.

The effectiveness of the AVG intervention was inconsistent. Another study conducted in the home setting reported that AVGs were ineffective in improving physical function [20], whereas studies in the hospital setting clarified the effectiveness of using AVGs as a supplementary tool for center-based, conventional cardiac rehabilitation programs [18,19]. The adherence rates and occurrence of adverse events showed similar trends in the identified studies. Regarding safety management, studies in the hospital setting controlled exercise intensity by monitoring the participants’ physiological changes during the intervention, whereas those in the home setting provided safety guidelines and instructions for adapting AVG play to each patient’s physical condition before the intervention. Furthermore, patients could call the research staff to ask questions during a given time period. Although myalgia and osteoarthritic knee pain occurred as musculoskeletal-related events in 2 studies, no cardiac-related adverse events were reported during the interventions. To manage patient adherence, the research staff conducted motivational calls or provided telephone guidance to each patient. The dropout rate seemed to be relatively low (0%-23%).

**Table 1:** Summary of study characteristics.

Study	Setting	Age, composition, and disease state	Active video game	Dose and duration	Outcome
Alves da Cruz et al, 2020 [18], cluster randomized controlled trial in Brazil	Hospital	N=27 48% female Age: 63.4 (12.71) years Status: CVD,^a^ CVD risk	Console: Xbox Kinect Program: Just Dance 2015 (warm-up), Shape Up (conditioning)	Period: 15 min (warm-up), 30 min, (conditioning) Frequency: Once Duration: 1 day Intensity: Based on heart rate reserve and rate of perceived exertion during intervention	Hemodynamics: systolic blood pressure, diastolic blood pressure, respiratory rate, oxygen saturation, heart rate, rate of perceived exertion
Jaarsma et al, 2020 [20], open-label randomized study in multiple countries	Home	N=605 29% female Age: 67 (12) years Status: HF^b^	Console: Nintendo Wii Program: Nintendo Wii Sports	Period: 30 min per day Frequency: 5 days a week Duration: 12 weeks Intensity: not reported	EC^c^: 6MWT^d^ Muscle function: unilateral isotonic heel-lift, bilateral isometric shoulder abductions, unilateral isotonic shoulder flexion EM^e^: Questionnaire Exercise SE^f^: Questionnaire PA^g^: single question
Klompstra et al, 2013 [21], case study in Sweden	Home	N=1 0% female Age: 74 years Status: HF	Console: Nintendo Wii Program: Nintendo Wii Sports	Period: 15 min Frequency: everyday Duration: 12 weeks Intensity: not reported	PA: accelerometer EC: 6MWT EM: questionnaire Exercise SE: questionnaire Perceived physical effort: Borg scale
Klompstra et al, 2014 [22], pilot study in Sweden	Home	N=32 31% female Age: 63 (19-88) years Status: HF	Console: Nintendo Wii Program: Nintendo Wii Sports	Period: 20 min per day Frequency: not recorded Duration: 12 weeks Intensity: not recorded	EC: 6MWT PA: accelerometer
Ruivo et al, 2017 [19], pilot randomized controlled trial in Ireland	Hospital	N=32 18.7% female Age: 59.9 (10.2) years Status: CVD	Console: Nintendo Wii Program: Nintendo Wii Sports	Period: 1-hour sessions Frequency: twice a week Duration: 6 weeks Intensity: based on heart rate from a precardiac rehabilitation test	EC: Bruce ramp protocol PA: accelerometer

^a^CVD: cardiovascular disease.

^b^HF: heart failure.

^c^EC: exercise capacity.

^d^6MWT: 6-minute walk test.

^e^EM: exercise motivation.

^f^SE: self-efficacy.

^g^PA: physical activity.

**Table 2:** Summary of intervention delivery and effectiveness across studies.

Study	Supervision	AVG^a^ alone/with other programs	Effectiveness	
			Results	Conclusions
Alves da Cruz et al, 2020 [18]	SV^b^	With cardiac rehabilitation program	Increased heart rate Increased respiratory rate Increased rate of perceived exertion	Greater heart rate, respiratory rate, and rate of perceived exertion were observed during and 5 min after the AVG session
Jaarsma et al, 2020 [20]	No SV	AVG alone	No change^c^ in exercise capacity, muscle function, exercise motivation, exercise self-efficacy, or PA^d^	AVG was safe and feasible in patients with heart failure Not effective in improving outcomes
Klompstra et al, 2013 [21]	No SV	AVG alone	Increased PA Increased exercise motivation Increased exercise self-efficacy No change in perceived physical effort	Further research is needed to generalize the results from the case study
Klompstra et al, 2014 [22]	No SV	AVG alone	Exercise capacity No change in PA	AVG has the potential to increase exercise capacity in patients with heart failure
Ruivo et al, 2017 [19]	SV	With conventional program	Increased PA Increased energy expenditure per body weight	Cardiac rehabilitation sessions with AVG are feasible and safe Significant improvement in PA and energy expenditure

^a^AVG: active video game.

^b^SV: supervision.

^c^After correction for baseline and confounders.

^d^PA: physical activity.

**Table 3:** Summary of adverse events and adherence across studies.

Study	Adverse events		Adherence		
	Safety management	Number of adverse events	Adherence management	Dropout rate, n (%)	Reasons for dropout
Alves da Cruz et al, 2020 [18]	Control exercise intensity	Cardiac, 0 Musculoskeletal, 0	NR^a^	0	NR
Jaarsma et al, 2020 [20]	Instruction for adapting active video games Phone consultation	Cardiac, 0 Musculoskeletal, 0	Motivational calls	71 (23%)	Medical-related issues Practical logistic reasons Refusal to continue Loss to follow-up Death
Klompstra et al, 2013 [21]	Safety guideline Phone call to heart failure nurse	Cardiac, 0 Musculoskeletal, 0	Remote guidance Follow-up visits	0	NR
Klompstra et al, 2014 [22]	Safety guideline Phone call to heart failure nurse	Cardiac, 0 Musculoskeletal, 1 (myalgia)	Telephone guidance	1 (3%)	Moved abroad for work
Ruivo et al, 2017 [19]	Playing active video games within individual target heart rate zones (55%-70%) Safety guideline Supervision at all times Monitoring by telemetry	Cardiac, 0 Musculoskeletal, 1 (osteoarthritic knee pain)	Motivational calls	1 (6.3%)	Returned to work

^a^NR: not reported.

## Discussion

### Overall Findings

This scoping review aimed to explore the possibility of applying commercially available AVGs to cardiac rehabilitation. Only 5 articles with commercially available AVGs were identified. All identified studies involved AVG interventions with a fixed time and frequency; however, exercise intensity was ill-defined in studies performed in the home setting. AVG interventions for patients with CVD may be safe and feasible regardless of the setting. On the other hand, some challenges seem to remain for the application of commercially available AVGs as therapeutic exercise tools in cardiac rehabilitation from the perspective of the balance between effectiveness and safety management, especially in terms of controlling exercise intensity.

### Effectiveness

Although 4 identified studies provided interventions involving AVGs in a given period to patients with CVD, the effectiveness of the interventions in improving physical function was inconsistent. Conventional cardiac rehabilitation programs recommend that therapeutic exercise be generally composed of a combination of 2 types of exercise: aerobic and resistance [23]. Aerobic exercise is defined as any activity that uses large muscle groups, can be maintained continuously, and is rhythmic in nature [24], whereas resistance exercise is expected to enhance the strength of major muscle groups by contracting muscles against external resistance [24]. It is essential for AVGs to require both types of exercise during play for adaptation to therapeutic exercise in cardiac rehabilitation. Regarding the game programs, sports-related programs that cause whole body movement during play, such as baseball and golf, were used. Several studies have reported the exercise intensity achieved during play for each game program in older people and patients after chronic stroke [25-27] but not in patients with CVD. Two randomized studies used an AVG as an intervention tool, but the results were inconsistent. An international multicenter study by Jaarsma et al [20] investigated the effects of access to a home-based AVG (Nintendo Wii) in patients with heart failure on submaximal aerobic exercise capacity, as assessed by the 6-minute walk test (6MWT), and muscle function, as measured by unilateral isotonic heel-lift, bilateral isometric shoulder abduction, and unilateral isotonic shoulder flexion. The treatment effects in the 6MWT were not significant at 3 months (4.3 m; 95% CI –6.9 to 15.5), 6 months (1.8 m; 95% CI –10.3 to 14), or 12 months (6.8 m; 95% CI –7.1 to 20.7) after correcting for baseline 6MWT results and confounders. Regarding muscle function, no outcomes were significantly improved except for left heel rise at 6 months. On the other hand, another study reported significant improvement in daily physical activity and related energy expenditure per body weight after a 6-week intervention compared with that in the control group [19]. In that study, Nintendo Wii was used as the supplementary tool for the center-based, conventional cardiac rehabilitation program. The results suggest that exercise induced by AVGs without supervision is not sufficient to improve physical function. From the perspective of exercise prescription, it is essential to clarify the exercise intensity of each game program in specific subjects. To implement more effective therapeutic exercises in home-based cardiac rehabilitation, further laboratory-based studies are needed in which exercise intensity is measured in patients with CVD while they play AVGs. Additionally, the AVG consoles used in the identified studies were Xbox Kinect and Nintendo Wii, neither of which provides external resistance to cause muscle contractions. Therefore, it might be better to use AVGs in combination with resistance training. A device that can detect muscle contractions as a signal for controlling AVGs also needs to be developed. Commercially available AVGs are designed to encourage players to engage in physical activity continuously; therefore, AVG-based exercise interventions in the home setting may be applicable to patients with CVD from the perspective of promoting safe physical activity.

### Safety Management

Regarding safety management, the identified studies prepared safety guidelines prior to the interventions. Patients could call an instructor or nurse at a given time in studies conducted in the home setting. In addition, in the identified studies, no cardiac-related adverse events were reported. The dropout rates were relatively low compared with those with conventional cardiac rehabilitation [28], partially because of the appealing design of commercially available AVGs such as the Nintendo Wii and Xbox Kinect for increasing motivation and long-term engagement to play the game. The challenges associated with center-based cardiac rehabilitation programs are low participation and high premature dropout rates [28-30]. A variety of barriers that can be characterized at 3 interrelated levelspatient, provider, and health care systemhave been reported in previous studies [6,31-33]. Older age, lower socioeconomic status, schedule conflicts, disinterest in attending a program, and comorbidities were included as barriers with respect to patients’ characteristics. Insufficient physician knowledge about the effectiveness of cardiac rehabilitation and inappropriate referrals to cardiac rehabilitation are often described as barriers at the provider level. System-level barriers to cardiac rehabilitation include transportation problems and the limited availability of cardiac rehabilitation programs for outpatients. Potential approaches to overcome these challenges include home-based cardiac rehabilitation and cardiac telerehabilitation [34-36]. Recent systematic reviews have concluded that home- and center-based cardiac rehabilitation have similar benefits in terms of clinical events, exercise capacity, and health-related quality of life among patients after myocardial infarction or coronary revascularization or among patients with heart failure [37,38]. Furthermore, another systematic review concluded that multidisciplinary or exercise-based cardiac telerehabilitation is safe and cost-effective and can be an alternative option to center-based cardiac rehabilitation in patients with coronary artery disease and chronic heart failure [35]. As AVG interventions seem to be safe and feasible for keeping patients with CVD active at home, home-based cardiac rehabilitation and cardiac telerehabilitation in conjunction with AVGs could help such patients achieve and maintain a more active lifestyle.

### Future Perspectives

AVGs are rapidly evolving with improvements in motion-sensing technologies, and 2 such AVG consoles, Nintendo Wii and Microsoft Kinect, were used as intervention tools for patients with CVD in the identified studies. Nintendo Wii, released in 2006, uses motion sensing technology in the primary controller to enable users to control game actions through their arm gestures. Nintendo Wii was the first commercially available video game console to induce body movement in the real world to manipulate objects or play sports in a virtual world. Following the Nintendo Wii, Microsoft launched Kinect in 2010 as a line of motion-sensing input devices. Microsoft Kinect, which was introduced to replace traditional game controllers, enables users to play games by using signals from whole-body gestures. A systematic review, published in 2016 [16] on the effectiveness of commercial AVGs for physical rehabilitation of motor function reported that almost 80% of included studies (n=126) used Nintendo Wii as the AVG console, which was a far higher rate than the rate at which Microsoft Kinect was used. The evolution seen in the past decade in motion-sensing systems to detect human body movement has accelerated the integration of commercial AVGs into therapeutic exercise for older people [10-13], patients after stroke [14], and patients with multiple sclerosis [39]. However, the application of AVGs in patients with CVD has yet to be addressed, partially because current AVG consoles do not induce resistance exercise. There could be 2 options to break through this barrier: the use of electromyographic signals and the development of a muscle contraction–induced controller. Some video games have introduced an electromyographic system to detect muscle contractions in the research setting [40,41] but none in the commercial setting. AVG consoles incorporating an electromyographic system in combination with a motion-sensing system could accelerate the application of AVGs in therapeutic exercise. Regarding the development of controllers that induce muscle contractions, Ring Fit Adventure, launched by Nintendo in 2017, is an action role-playing game for the Nintendo Switch that consists of 2 physical components as accessories: The Leg Strap and Ring-Con. The Leg Strap affixes the original controller to the user’s thigh to detect leg movement, and Ring-Con is a ring that includes a strain sensor that can detect the bending of the ring by using the original controller as a logger. These accessories enable users to engage in both aerobic and resistance exercise during gameplay. Isometric resistance exercises usually require breath-holding, which elevates blood pressure. Speech recognition systems have the potential for safety management by not requiring users to hold their breath while performing resistance exercises even remotely. However, to our knowledge, no studies have investigated exercise intensity during gameplay using Ring Fit Adventure, which could be the first step in clarifying exercise intensity for its application as a tool in safe therapeutic exercises as a part of cardiac rehabilitation. Concurrently, the feasibility of the combined use of AVGs and other devices for monitoring muscle contractions should be investigated to enable safe therapeutic exercise in home-based cardiac rehabilitation.

Subjects in identified articles in this review spanned a wide range of generations, including older generations. With increasing age, digital divide—the gap between individuals who have access to modern information and communication technology, including digital devices, and those who lack access—should be considered. Older generations usually do not have the opportunity or knowledge to use digital devices and therefore could not utilize them properly. In an identified study investigated in the home setting [22], the instructor demonstrated the AVG play at the patients’ home and also conducted a 1-hour introduction session at the hospital. Proper introduction of AVGs is essential for its application in therapeutic exercise, especially in older adults.

### Limitations

Commercially available AVGs could potentially be applied to cardiac rehabilitation; however, the evidence obtained in this review should be interpreted with caution because research in this field is rapidly growing. In addition, this scoping review excluded studies that used commercially available AVG consoles with programs designed for research, which could offer hints for the next step toward the smooth application of commercially available AVGs in cardiac rehabilitation.

### Conclusions

The results of this scoping review suggest that evidence remains lacking for the application of commercially available AVGs in cardiac rehabilitation for patients with CVD in both laboratory-based and clinical studies. Commercially available AVGs may be suitable as a tool for promoting an active lifestyle in patients with CVD even in the home setting. However, commercially available AVGs should be used with caution as an alternative to therapeutic exercise. The need for remotely accessible cardiac rehabilitation programs, including therapeutic exercises, is increasing because of the aging population. Further studies are needed to investigate the appropriate frequency, intensity, and duration of time of commercially available AVGs for effective therapeutic exercises in patients with CVD. In addition, the development of devices that can detect muscle contractions when AVGs are played is recommended for the addition of resistance exercise and institution of more effective therapeutic exercises.

## Appendix

Multimedia Appendix 1 Literature search terminology and number of records included from databases.

## Abbreviations

AAU: accelerometer arbitrary unit
AVG: active video game
CVD: cardiovascular disease
PRISMA-ScR: Preferred Reporting Items for Systematic Reviews and Meta-Analyses Extension for Scoping Reviews
6MWT: 6-minute walk test
