# Supplementary material for: The Potential Application of Commercially Available Active Video Games to Cardiac Rehabilitation: Scoping Review
Source: JMIR Serious Games. 2022 Mar 18;10(1):e31974. doi: 10.2196/31974 (PMC8976248; doi:10.2196/31974)
Supplement: Multimedia Appendix 1 [file games_v10i1e31974_app1.docx]

**Appendix 1. Literature Search Terminology and Number of Records Included from Databases**

| **Search Terms** | |
| --- | --- |
| (“gamificatio*” OR “exergam*” OR “virtual reality” OR “active video gam*” OR “active computer gam*”) AND (“cardiovascular disease” OR “heart disease” OR “heart failure” OR “myocardial infarction” OR “myocardial ischemia” OR “angina pectoris” OR “myocardial revascularization” OR “coronary artery bypass”) | |
| **Database** | **Number of Records** |
| CINAHL | 45 |
| MEDLINE | 84 |
| PubMed | 73 |
| SPORTDiscus | 13 |
| Hand Search | 8 |
| **Total** | **223** |

CINAHL, Cumulative Index to Nursing and Allied Health Literature
